# Tn5 transposase and tagmentation procedures for massively scaled sequencing projects

**DOI:** 10.1101/gr.177881.114

**Published:** 2014-12

**Authors:** Simone Picelli, Åsa K. Björklund, Björn Reinius, Sven Sagasser, Gösta Winberg, Rickard Sandberg

**Affiliations:** 1 Ludwig Institute for Cancer Research, 171 77 Stockholm, Sweden;; 2 Department of Cell and Molecular Biology, Karolinska Institutet, 171 77 Stockholm, Sweden

## Abstract

Massively parallel DNA sequencing of thousands of samples in a single machine-run is now possible, but the preparation of the individual sequencing libraries is expensive and time-consuming. Tagmentation-based library construction, using the Tn5 transposase, is efficient for generating sequencing libraries but currently relies on undisclosed reagents, which severely limits development of novel applications and the execution of large-scale projects. Here, we present simple and robust procedures for Tn5 transposase production and optimized reaction conditions for tagmentation-based sequencing library construction. We further show how molecular crowding agents both modulate library lengths and enable efficient tagmentation from subpicogram amounts of cDNA. The comparison of single-cell RNA-sequencing libraries generated using produced and commercial Tn5 demonstrated equal performances in terms of gene detection and library characteristics. Finally, because naked Tn5 can be annealed to any oligonucleotide of choice, for example, molecular barcodes in single-cell assays or methylated oligonucleotides for bisulfite sequencing, custom Tn5 production and tagmentation enable innovation in sequencing-based applications.

The unprecedented increase in sequencing machine capacity (Metzker 2009) has led to improved throughput and lowered cost for DNA and RNA sequencing (RNA-seq) applications. For example, expression profiles from hundreds of different single cells can be generated in a single lane of an Illumina HiSeq 2000 (Hashimshony et al. 2012; Islam et al. 2014; Jaitin et al. 2014). While the sequencing costs have been drastically decreasing, the throughput and costs of library preparation are now the limiting factor. This represents a severe constraint in many next-generation sequencing–based projects, especially now that the importance of obtaining expression profiles from hundreds or thousands of single cells is more fully appreciated (Eberwine et al. 2014; Sandberg 2014). Indeed, improved automation and lowered reagent costs in sequence library generation are needed to harness the full potential of current sequencing technology and to make it affordable for any laboratory around the world.

A great advancement in library preparation was the introduction of a hyperactive variant of the Tn5 transposase that mediates the fragmentation of double-stranded DNA and ligates synthetic oligonucleotides at both ends in a 5-min reaction (Adey et al. 2010). Wild-type Tn5 transposon is a composite transposon in which two near-identical insertion sequences (IS50L and IS50R) are flanking three antibiotic resistance genes (Reznikoff 2008). Each IS50 contains two inverted 19-bp end sequences (ESs), an outside end (OE) and an inside end (IE). However, wild-type ESs have a relatively low activity and were replaced in vitro by hyperactive mosaic end (ME) sequences. A complex of the transposase with the 19-bp ME is thus all that is necessary for transposition to occur, provided that the intervening DNA is long enough to bring two of these sequences close together to form an active Tn5 transposase homodimer (Reznikoff 2003). Transposition is a very infrequent event in vivo, and hyperactive mutants were historically derived by introducing three missense mutations in the 476 residues of the Tn5 protein (E54K, M56A, L372P), which is encoded by IS50R (Goryshin and Reznikoff 1998). Transposition works through a “cut-and-paste” mechanism, where the Tn5 excises itself from the donor DNA and inserts into a target sequence, creating a 9-bp duplication of the target (Schaller 1979; Reznikoff 2008). In current commercial solutions (Nextera DNA kits, Illumina), free synthetic ME adaptors are end-joined to the 5′-end of the target DNA by the transposase (Adey et al. 2010). Although the data quality and reproducibility are high when using Nextera kits, the cost of these commercial reagents limits its use to small- or medium-sized projects in most research projects, and the preannealing of adaptor oligos precludes the development of novel applications.

Here, we report a simple procedure for producing high-quality, tagmentation-ready Tn5 transposase and demonstrate all the necessary steps for efficient generation of sequencing libraries. Through the generation of more than 100 sequencing libraries, we demonstrate how to modulate the average size of the tagmented fragments using different buffer compositions, and we show how to obtain libraries from subpicogram amounts of DNA. Finally, we demonstrate that in-house and commercial Tn5 generate single-cell RNA-seq libraries of similar quality and that in-house Tn5 can be used to lower costs in present and novel massively parallel, sequencing-based applications (Adey and Shendure 2012; Islam et al. 2014).

## Results

### Production of active Tn5 transposase

**Figure 1.**
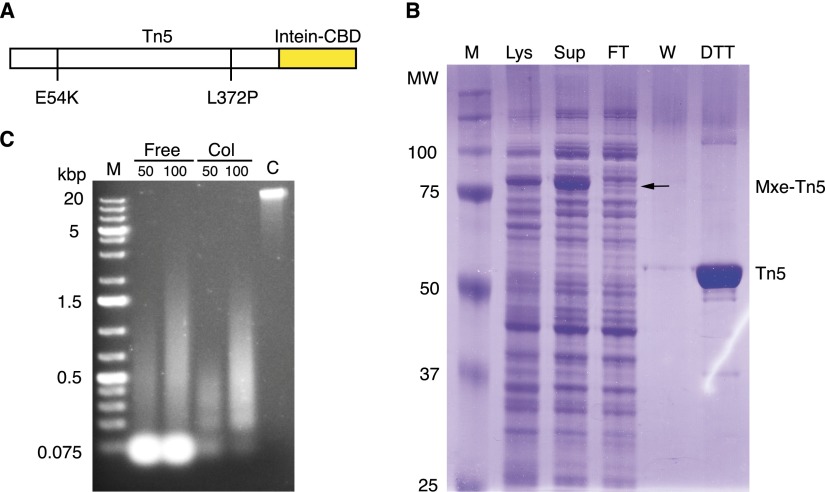
Production and purification of tagmentation-ready Tn5. (*A*) Illustration of the Tn5–intein fusion construct. (*B*) 10% SDS-PAGE run with crude lysate (Lys), supernatant (Sup), flow-through (FT), wash (W), and DTT eluted fractions (DTT) from the chitin column. The expected sizes of the Tn5–intein fusion protein (Mxe-Tn5) and cleaved full-length Tn5 (Tn5) are indicated on the *right*; marker molecular weights in kDa, to the *left*. (*C*) Agarose gel (1% Type LE, in TAE buffer) demonstrating tagmentation of 50 or 100 ng high MW calf thymus DNA with 1 μL of Tn5 prepared by annealing in solution (Free), on chitin columns (Col), or with no Tn5 as a negative control (C). On-column annealing removed excess oligonucleotides present after solution annealing.

We cloned a hyperactive *Tn5* allele harboring the classical E54K and L372P mutations (but wild-type at M56) into pTBX1 (pTXB1-Tn5) to add a C-terminal intein tag and a chitin-binding domain (CBD) (Fig. 1A; Supplemental Fig. 1). We found that the additional mutations (Y64I, K200R, and S303G) that were introduced in US patent 5,965,443 (Reznikoff and Goryshin 1996) yielded a nearly inactive protein (data not shown). Previously, Tn5 expression from the pTYB4 (NEB) vector was reported (Bhasin et al. 1999), in which the Tn5 CDS is C-terminally fused to the *Saccharomyces cerevisiae* VMA1 intein with an additional glycine before the catalytic cysteine to achieve efficient cleavage with DTT. Expressing Tn5 in the pTXB1 vector had several advantages: First, the *Mycobacterium xenopi* GyrA intein is half the size of the *S. cerevisiae* VMA1 intein, which facilitates higher expression levels of the fusion protein without formation of inclusion bodies; second, the natural C-terminal isoleucine of Tn5 supports efficient intein cleavage in this vector. The T7 expression system was found to suppress production of detectable amounts of Inhibitor (Inh; data not shown). Expression of the *M. xenopi* GyrA-Tn5 fusion protein was induced with isopropyl β-D-1-thiogalactopyranoside (IPTG), cell DNA in the crude lysate was removed by polyethylenimine (PEI) precipitation, and the cleared lysate was loaded on a chitin column. We observed that oligonucleotides containing the hyperactive ME sequences could be annealed to Tn5 already on the columns (so that excess unannealed oligonucleotides were discarded through the column) or later in solution. Tn5 was released from the column by DTT-induced cleavage of the intein–CBD tag (Fig. 1B). We initially assessed the activity of the assembled Tn5 through its ability to tagment high-molecular-weight (HMW) DNA into 400- to 500-bp fragments, with a representative gel shown in Figure 1C.

### Development of efficient tagmentation reactions

**Figure 2.**
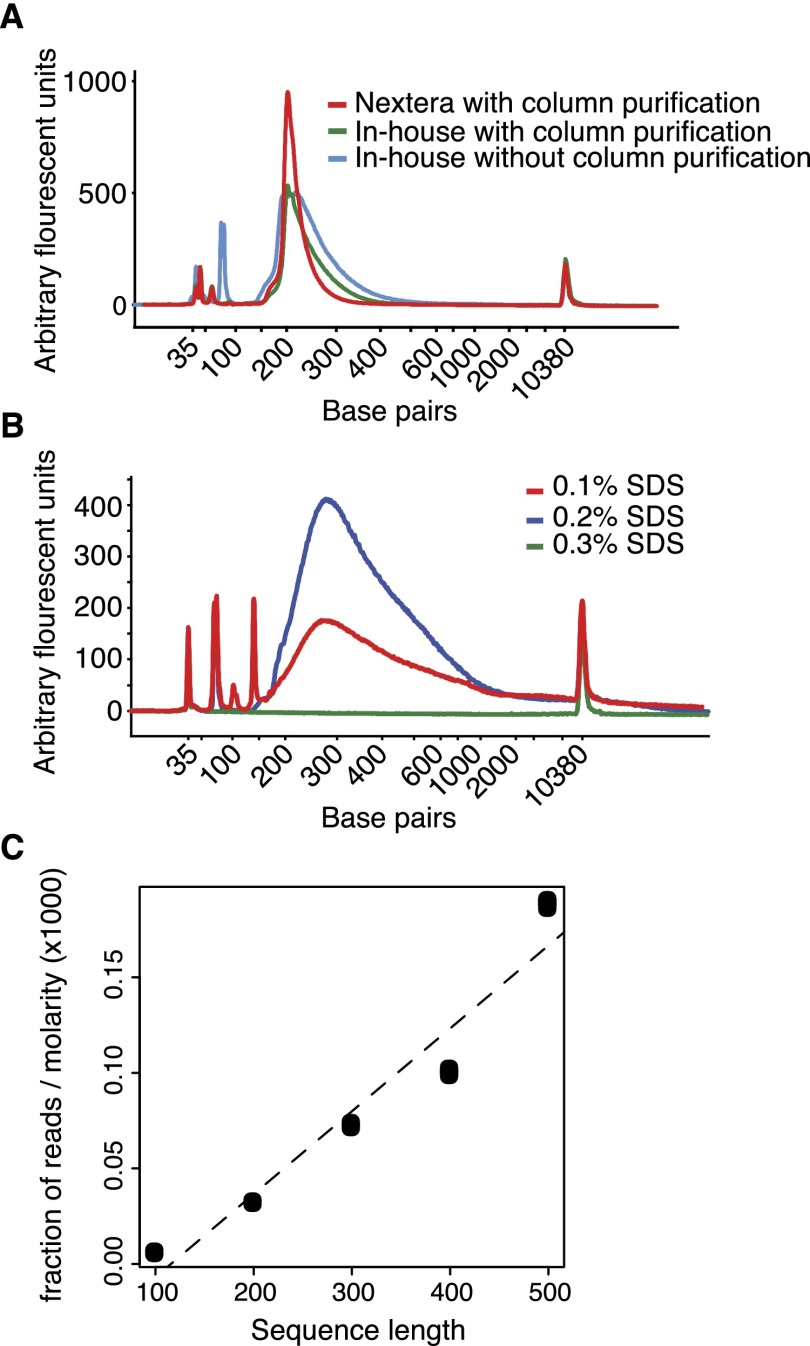
Efficient and robust tagmentation reactions. (*A*) Bioanalyzer electropherograms of tagmented DNA libraries using Nextera (with column purification), in-house Tn5, and buffers with and without column purification. (*B*) Bioanalyzer electropherograms of tagmented DNA libraries generated with in-house Tn5 that were stripped with different amounts of SDS. (*C*) Fraction of sequenced reads mapping to DNA ladder sequences of varying lengths. The fraction of reads was adjusted for molarity of DNA ladder sequence, as determined with Bioanalyzer (Agilent).

To find robust conditions for an efficient tagmentation reaction and to validate our in-house-produced Tn5 transposase, we performed a large set of experiments with different reaction buffers and variations. The initial Epicentre kit (for Illumina sequencing) used a Tris-based buffer with a pH of 8.0 at 25°C. Although sufficient for tagmentation, its pH will decrease at increased temperatures, and we instead chose a TAPS-based buffer with a stable pH of 8.5 in the range 25°C–55°C. The magnesium chloride concentration in the TAPS buffer (5 mM final concentration) was important, as higher concentrations gave progressively lower yields after enrichment PCR. We also lowered the reaction volume to 20 μL and replaced column purification with SDS or water. Finally, we substituted the undisclosed DNA polymerase used in the final enrichment PCR with the GC-tolerant KAPA HiFi DNA polymerase (KAPA Biosystems). For comparison, tagmentation and enrichment PCR were performed using all reagents from the Nextera kit (Illumina). In this case, purification after tagmentation was done as suggested by Illumina, using the DNA Clean & Concentrator-5 kit from Zymo Research. We uncovered that robust tagmentation occurs when stripping the transposase from DNA with 0.1% SDS and performing the adaptor-ligated enrichment PCR with KAPA HiFi DNA polymerase in the same tube, without the standard column-based purification (Fig. 2A). Besides being a simplification of the workflow, this has the additional advantage of increasing the sample throughput. Interestingly, comparable results were obtained with the Nextera kit when adding water after tagmentation and proceeding directly with the PCR, indicating that the subsequent incubation at 72°C (gap filling) probably suffices to release the transposase from the DNA. Conversely, our in-house Tn5 necessitates a stripping buffer with at least 0.1% SDS. Fine-tuning the concentration of SDS, we found that 0.2% SDS was optimal for stripping, resulting in a higher yield after PCR, while increasing the concentration of SDS to 0.3% led to a complete failure of the PCR amplification (Fig. 2B).

### Tagmentation of short DNA fragments

For RNA-seq applications, it is important to determine whether tagmentation efficiency depends on DNA fragment length, as complementary DNA (cDNA) have heterogeneous lengths. Earlier work on Tn5 (Adey et al. 2010) observed that transposon-based libraries had a minimum insert length of ∼35 bp, but it has not been demonstrated whether shorter DNA fragments would be less efficiently captured in tagmentation-derived sequencing libraries. We generated sequencing libraries based on Tn5 tagmentation of a DNA ladder of short fragments and found that the fraction of reads aligning to fragments correlated (Spearman correlation = 0.8) with sequence length (Fig. 2C). We concluded that tagmentation of fragments is fairly unbiased with respect to length, even down to 100-bp fragments.

### Validation of tagmentation reactions and Tn5 in single-cell RNA-seq

**Figure 3.**
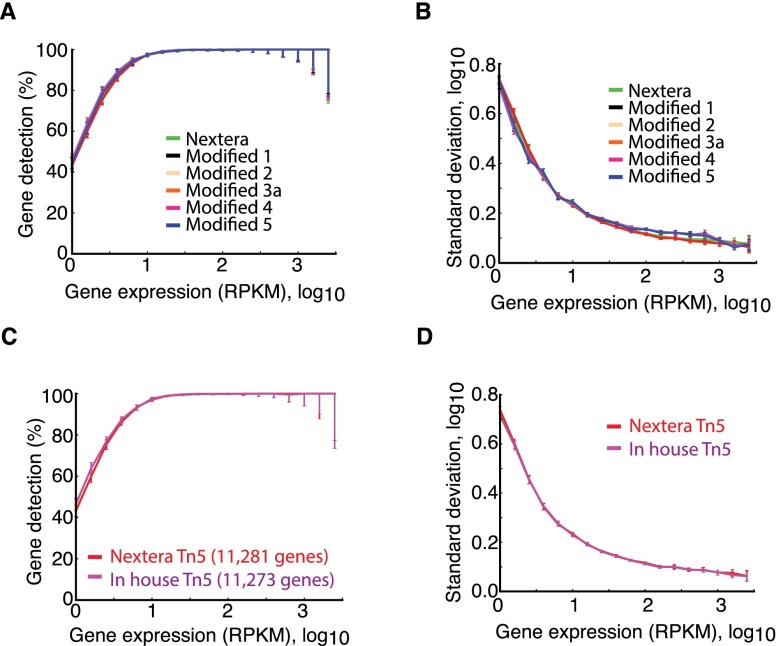
Comparable single-cell gene expression profiles with in-house Tn5. (*A*) Percentage of genes reproducibly detected in replicate cells, binned according to expression level. We performed all pairwise comparisons within replicates for the libraries generated with Nextera protocol or five modified protocols exchanging different procedure components (Supplemental Table 1) and report the mean and 90% confidence interval. (*B*) Standard deviation in gene expression estimates within replicates for Nextera or five variant protocols (as in *A*) in bins of genes sorted according to expression levels. Error bars, SEM (*n* ≥ 4). (*C***,***D*) Comparison, as in *A* and *B*, for sequence libraries generated with identical reaction buffers (Supplemental Table 1) but with either produced or commercial Nextera Tn5 transposase.

To validate libraries generated with our Tn5 and reaction conditions, we generated RNA-seq libraries from 5 ng cDNA from MEF and C2C12 cell lysate splits generated with Smart-seq2 (Picelli et al. 2013). We carried out the tagmentation with both commercial (Nextera, Illumina) and in-house Tn5 in a variety of reaction conditions (for a detailed description of all modifications, see Supplemental Table 1). We sequenced three or more replicate libraries generated under each condition and compared the genome-wide expression profiles. Analyses of aligned RNA-seq data demonstrated that both the sensitivity (Fig. 3A) and the accuracy (Fig. 3B) of the expression estimates were essentially identical in libraries generated with commercial or our in-house developed reaction conditions. We also observed very similar sequencing library characteristics in terms of aligning frequencies to the genome and transcriptome, mismatch frequencies, and sequence bias for tagmentation (Supplemental Figs. 2, 3). A direct comparison between the commercial and our in-house produced Tn5 (keeping all other conditions constant, see Supplemental Table 1) showed that the two sequencing libraries had identical gene expression sensitivity (Fig. 3C) and accuracy (Fig. 3D). Therefore, we conclude that our in-house Tn5 and reaction conditions generate sequencing libraries of similar quality as the commercial solution, but with improved throughput so that it can be more efficiently used for single-cell RNA-seq applications (Picelli et al. 2013, 2014).

### Flexible tagmentation on low starting cDNA amounts

**Figure 4.**
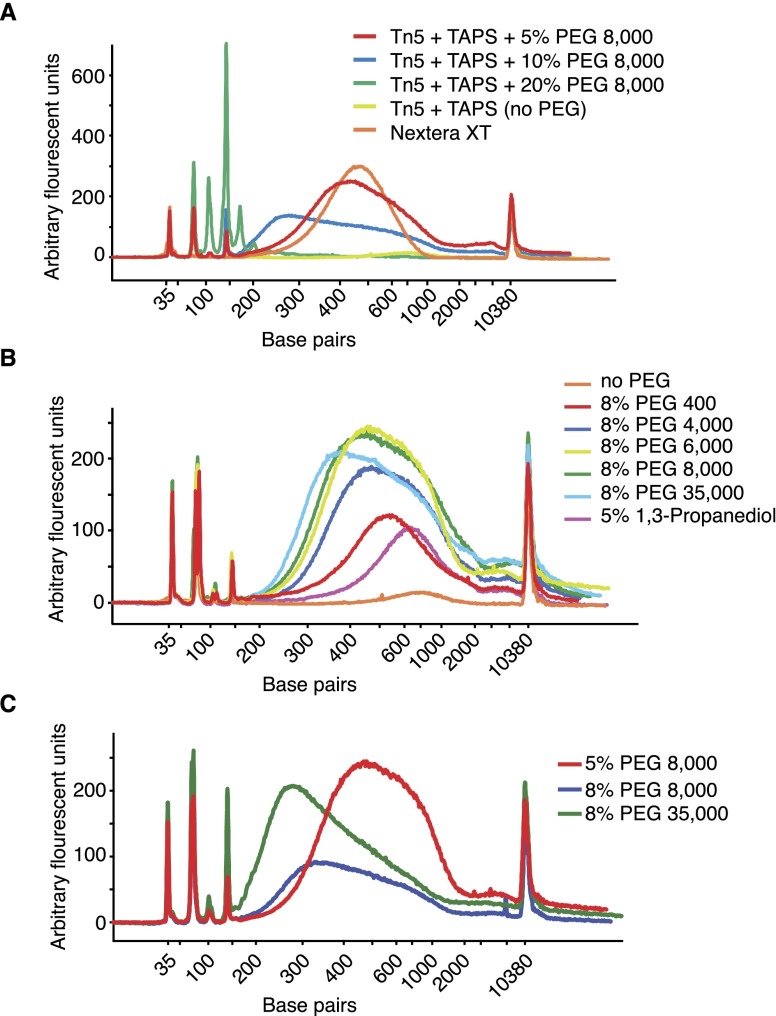
Tagmentation reactions with molecular crowding agents. (*A*) Bioanalyzer electropherograms of tagmented DNA from reactions with different concentrations of PEG 8000 or no PEG or using Nextera XT reaction conditions. (*B*) Bioanalyzer electropherograms of tagmented DNA using PEG polymers of different MWs. (*C*) Representative Bioanalyzer electropherograms of tagmented DNA used to optimize the concentration of PEG 8000 in tagmentation reactions and to demonstrate how tagmented fragment size can be modulated by PEG amounts. (*A–C*) All reactions were performed on 100 pg HEK293T cell cDNA together with 12 (*A*) or 13 (*B*,*C*) cycles of enrichment PCR.

When starting from 1 ng cDNA or higher, we obtained good quality libraries using a tagmentation buffer containing TAPS, magnesium chloride, and dimethylformamide (DMF). However, when lowering the input to picogram levels, we found that a different buffer and specific additives were needed for successful tagmentation (Table 1; Fig. 4A). In particular, it was necessary to replace DMF with a crowding agent such as polyethylene glycol (PEG). A broad range of PEG polymers with different MW were evaluated: PEG 400, PEG 4000, PEG 6000, PEG 8000, and PEG 35,000. The optimum was reached with a final PEG concentration of 8% in the tagmentation reaction, while there were no major differences with respect to sequence read quality and quantity between PEG 4000, PEG 6000, or PEG 8000. PEG 35,000 produced low-quality libraries, and PEG 400 was ineffective, especially when starting from < 500 pg. PEG concentrations > 10% led to broader peaks and lower yields, while at < 5% no beneficial effect was observed (Fig. 4A,B). We also observed that, while the data quality was comparable when using various PEG polymers, the average size of the libraries after enrichment PCR was inversely related to the polymer length, with the longest polymers giving the libraries with the shortest average size (Fig. 4C). This is an indirect confirmation that a more efficient tagmentation is obtained through a reduction of the available volume in which the transposase can operate.

**Table 1.**
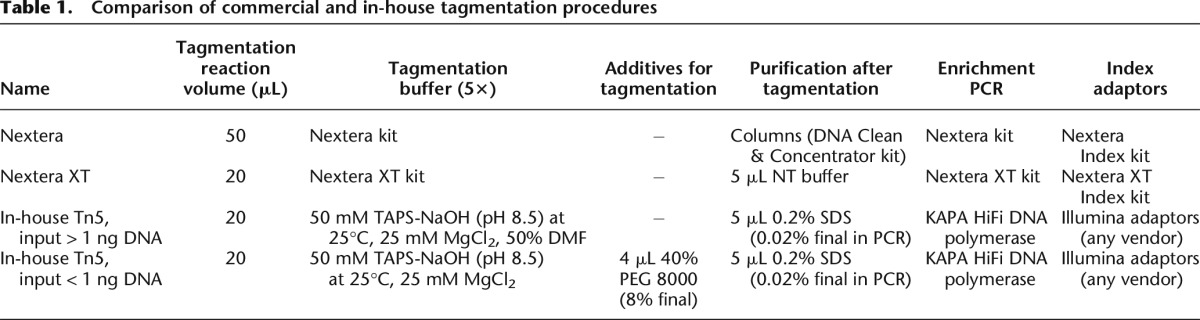
Comparison of commercial and in-house tagmentation procedures

### Tagmentation of subpicogram amounts of cDNA

**Figure 5.**
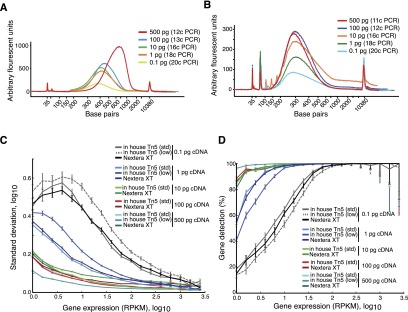
Lower limit of starting cDNA for tagmentation libraries. (*A*,*B*) Bioanalyzer electropherograms of tagmented DNA from reactions using 500 pg to 0.1 pg cDNA using either Nextera XT (*A*) or in-house Tn5 and reaction conditions including TAPS buffer and 8% PEG 8000 (*B*). (*C*) Technical variability in transcriptome libraries generated with Nextera XT or in-house buffers (std, standard Tn5 amounts; low, 1/100th Tn5 amount). Mean and standard deviation in gene expression estimates within replicates per protocol and in bins of genes sorted according to expression levels. Error bars, SEM (*n* ≥ 3). (*D*) Sensitivity in transcriptome libraries generated with Nextera XT or in-house buffers (std, standard Tn5 amounts; low, 1/100th Tn5 amount). We report the mean percentage of genes reproducibly detected in replicate experiments (all pairwise comparisons per protocol) and binned according to expression level, with 90% confidence intervals.

In order to test the lower limit of input DNA required for tagmentation, we generated libraries from 500, 100, 10, 1, and 0.1 pg of double-stranded DNA. We successfully obtained RNA-seq libraries with both commercial reagents (Fig. 5A) and in-house Tn5 and conditions (Fig. 5B) from all starting cDNA amounts. Computational analyses of the mapped reads identified no systematic differences in terms of sensitivity (Fig. 5C) or accuracy in expression level estimates (Fig. 5D) between commercial or in-house reagents, although the sensitivity, accuracy, and library complexity (Supplemental Fig. 4) declined at 1 and 0.1 pg input cDNA levels for both protocols (Fig. 5C,D). Additionally, we observed that a larger percentage of reads became unmappable (e.g., adapter artifacts) in libraries made with in-house Tn5 from only 1 or 0.1 pg cDNA (Supplemental Fig. 5), likely the result of overtagmentation of DNA due to highly excessive Tn5 amounts. To test this hypothesis, we generated additional libraries utilizing only a 1/100th of the Tn5 amount from 500, 1, and 0.1 pg of cDNA. Analyses of these libraries demonstrated much-improved mapping characteristics (Supplemental Fig. 4) without systematic effects on RNA-seq profile characteristics (Fig. 5C,D).

## Discussion

In this study, we describe a simple procedure for the production of in-house Tn5 transposase as a high-quality, yet inexpensive, alternative to the currently available commercial kits (Nextera and Nextera XT, Illumina). The availability of custom Tn5 transposase protein opens up innovation in both protocol design and applications. For example, Tn5 produced according to this procedure was successfully used to develop a molecular counting method for single-cell gene expression analyses (Islam et al. 2014) and can be employed for bisulfite sequencing applications (Wang et al. 2013). For such applications it is important to anneal custom oligonucleotides (precluding the use of commercial Tn5 that comes preannealed to their proprietary oligonucleotides).

Additionally, we developed efficient procedures for tagmentation of DNA that will allow for massively scaled sequencing projects. Nonspecific polymers, such as PEG, act as “crowding agents” in DNA and RNA ligation reactions (Zimmerman and Pheiffer 1983). When high amounts of macromolecules (e.g., proteins and polymers) are added to a solution, they sequester water (i.e., the excluded volume effect), lowering molecular diffusion rates and ultimately increasing the efficiency of biological reactions (Zimmerman and Pheiffer 1983). Using PEG as crowding reagent, we were able to generate high-quality sequencing libraries from as little as 0.1 pg of DNA with in-house Tn5 and commercial Tn5. Limited trials with other crowding agents such as Ficoll 70 (MW ≈ 70,000), a high molecular weight polymer formed by copolymerization of sucrose with epichlorohydrin, gave negative results and were not pursued further.

Although the Tn5 transposase requires magnesium ions as cofactor in the tagmentation reaction, the synaptic complex of Tn5 with double-stranded DNA was crystallized with two manganese ions in the active site (Lovell et al. 2002). However, we failed to generate libraries when replacing magnesium for manganese (in the form of MnCl_2_), indicating that magnesium is critical for tagmentation.

In this study we developed successful procedures for the production of high-quality Tn5, and we formulated an efficient tagmentation protocol that can be used for standard tagmentation, single-cell RNA-seq applications and even for minute input amounts of DNA. We did not notice reduced performance using these custom protocols, with the advantage of making Tn5 fully accessible to the research community, entirely relying on off-the-shelf reagents. This opens up novel and flexible tagmentation-based applications and will accelerate biological discovery by drastically reducing the cost of DNA-sequencing library construction.

## Methods

### Tn5 production

The pBam1 cloning vector was a generous gift from Dr. E. Martínez-García (Martínez-García et al. 2011). The hyperactive *Tn5* allele in this vector carries the classical E54K and L372P mutations but is wild type with respect to M56. The Tn5 coding sequence in pBam1 was PCR adapted for cloning into the pTXB1 vector (NEB) at the XbaI and LguI sites using the following primers: XBATN5, 5′-ACTCGTCTAGAAATAATTTTGTTTAACTTTAAGAAGGAGA-3′; TN5TER, 5′-gattttaatgccctgcgccatc-3′.

This amplicon includes the T7 promoter sequence from pBamI and fuses the terminal isoleucine of Tn5 to the catalytic cysteine of the *M. xenopi* GyrA intein, which allows precise excision of the Tn5 protein from the intein–CBD fusion partner without additional amino acids. While all previous protocols have used Tn5–intein–CBD fusion vectors with the *S. cerevisiae* VMA1 intein-CBD (MW 59226, pI 7.97), we find that the smaller size (MW 29665) and lower pI (pI 5.54) of the *M. xenopi* GyrA intein may improve the solubility of the corresponding Tn5 fusion protein, thus alleviating problems with inclusion body formation that we have observed using the larger intein vectors.

Six pTXB1 clones were fully sequenced and transformed into C3013 cells (NEB) for production. One liter of culture was grown at 37°C to A600 = 0.9. The culture was then chilled to 10°C, and IPTG was added to 0.25 mM. After continued growth for an additional 4 h at 23°C, the culture reached A600 3.0 and yielded 3–4 gr of cells, which were frozen at −70°C overnight and subsequently lysed by sonication in 80 mL HEGX (20 mM HEPES-KOH at pH 7.2, 0.8 M NaCl, 1 mM EDTA, 10% glycerol, 0.2% Triton X-100), with complete (Roche) protease inhibitors. Sonication was carried out for 10–12 cycles of 45–50 bursts with 50% duty cycle at output 7 on a Branson sonicator with a 10-mm tip with intermittent cooling in an ice–salt mixture using a stainless steel beaker. The lysate was pelleted in the Beckman JA17 rotor at 15,000 rpm for 30 min at 4°C. To the supernatant, 2.1 mL 10% neutralized PEI (Sigma P3143) was added dropwise on a magnetic stirrer, and the precipitate was removed by centrifugation at 12,000 rpm for 10 min at 4°C in the Beckman JA17 rotor. The introduction of the PEI precipitation step effectively reduced the fraction of *Escherichia coli* reads in our libraries.

The supernatant was loaded on a 10-mL chitin column (NEB) at 0.4 mL/min in HEGX with complete protease inhibitors. The column was washed at 0.2 mL/min with 200–300 mL HEGX, following which 24 mL HEGX, 100 mM DTT was added to the top of the column bed. Then 11-mL buffer was drained out of the column. The column was left closed for 36–48 h at 4°C to effect cleavage of Tn5 from the intein. The first 11 mL eluted contained 70%–90% of the Tn5. Elution was done in 1 mL aliquots, and the protein concentration was tested using a Bradford assay (Bio-Rad protein assay). One microliter of each fraction was added to 50 μL of assay solution, and the fractions with the strongest blue color were pooled and dialyzed versus two changes of 1 liter of 2× Tn5 dialysis buffer (100 HEPES-KOH at pH 7.2, 0.2 M NaCl, 0.2 mM EDTA, 2 mM DTT, 0.2% Triton X-100, 20% glycerol). A typical elution profile is shown in Figure 1B. After dialysis, the protein concentration was measured using a Nanodrop spectrophotometer (Thermo Scientific) and was used directly for transposome assembly if the absorbance was about A280 = 3.0 or higher. If lower, centrifugal concentration can be done using Amicon Ultracel 30 centrifugal filters (Millipore) at 3000 rcf at 4°C for 20- to 25-min periods with intermittent mixing until the needed Tn5 concentration for assembly is reached.

### Transposome assembly in solution

Assembly of Tn5 with preannealed Mosaic End double-stranded (MEDS) oligonucleotides can be performed in solution (see below and Reznikoff 2008) by mixing 0.125 vol of a 100 μM equimolar mixture of preannealed Tn5MEDS-A and Tn5MEDS-B oligonucleotides in TE, 0.4 vol 100% glycerol, and 0.12 vol 2× Tn5 dialysis buffer and finally, 0.36 vol Tn5 at A280 = 3.0, i.e., 1.85 mg/mL (Tn5 MW = 53300, Eμ = 86525, A280 = 1.000 = 0.616 mg/mL = 11.56 μM). The mixture is incubated for 60 min at room temperature (RT) and then stored at −20°C. Residual unbound MEDS can then be removed from the subsequent tagmentation reactions using DNA Clean & Concentrator (Zymo Research) spin columns or using Agencourt AMPure XP beads (Beckman Coulter).

Unassembled Tn5 (at 3.0 OD280) can be stored at −20°C as a 55% glycerol stock, after addition of 1.1 vol 100% glycerol and 0.33 vol of 2× Tn5 dialysis buffer to the dialyzed Tn5 preparation. This stock can be used directly for Tn5 transposome assembly by adding 0.143 vol 100 μM Tn5MEDS-A/B oligonucleotides and proceeding as above. For use with the Illumina system, the oligonucleotide sequences are as follows: Tn5MErev, 5′-[phos]CTGTCTCTTATACACATCT-3′; Tn5ME-A (Illumina FC-121-1030), 5′-TCGTCGGCAGCGTCAGATGTGTATAAGAGACAG-3′; and Tn5ME-B (Illumina FC-121-1031), 5′-GTCTCGTGGGCTCGGAGATGTGTATAAGAGACAG-3′.

For other applications, such as methylome sequencing (Wang et al. 2013), the single-stranded extension outside the 19-bp ME sequence may be chosen accordingly.

### On column transposase assembly

MEDS oligonucleotides are also bound by Tn5 protein while C-terminally attached to the chitin matrix through its *M. xenopi* GyrA–CBD fusion partner. This significantly facilitates the large-scale production of active transposase. In addition, the on-column assembly of transposase eliminates excess unbound MEDS oligonucleotides, which may interfere with sequence library construction, particularly from single cells. The cleared lysate after PEI precipitation is loaded on the chitin column, and the column is washed with 20 vol HEGX buffer as described above. For a 10-mL chitin column, 500 nmol of the mixed Tn5MEDS-A/-B oligonucleotides is added to the column in 12 mL degassed HEGX buffer. The column is left at room temperature overnight and then washed with 20 vol HEGX to remove free MEDS. After release from the chitin column with DTT (see above), the Tn5-MEDS complexes are dialyzed against Tn5 dialysis buffer (as described above) to remove excess DTT and adjusted to 12.5 μM concentration in 50% glycerol and 1× Tn5 dialysis buffer.

From a 1-L culture of induced 3013 cells, one may expect to recover ∼4 g of cell pellet, which yields at least 15 mg Tn5-GyrA-CBD, equivalent to ∼280 nmol, which is equal to the reported binding capacity of a 10-mL chitin column. Since the desalted synthesis yields of the oligonucleotides annealed to the Tn5 at the 1-mM scale is between 100 and 250 nmol (Sigma-Aldrich), it is necessary to plan oligonucleotide synthesis orders accordingly.

### Assays of transposase activity

Tn5 reactions were assembled by mixing 14 μL H_2_O, 4 μL 5× TAPS-MgCl_2_-PEG 8000 or 5× TAPS-DMF, 1 μL target DNA at 50 ng/μL, 1 μL of the Tn5, preassembled with A/B-MEDS oligonucleotides at 12.5 mM. Buffers used were as follows: 5× TAPS-PEG 8000 (50 mM TAPS-NaOH at pH 8.5 [RT], 25 mM MgCl_2_, 40% PEG 8000) or 5× TAPS-DMF (50 mM TAPS-NaOH at pH 8.5 [RT], 25 mM MgCl_2_, 50% DMF). For library production, the reactions were incubated for 7 min at 55°C, and then 5 μL 0.2% SDS (final 0.02%) was added and Tn5 was inactivated for 7 min at room temperature or 55°C. Typically 5 μL of the Tn5 reactions was used directly in the subsequent enrichment PCR amplification of libraries for the Illumina sequencers. Successful libraries were also generated using only the index adaptors as they contain the entire sequence of the PCR primers (FC-121-1012 and FC-121-1011).

For testing transposase activity, reactions were set up as above, with 50 ng HMW DNA as substrate. To stop the reactions, 0.5 μL proteinase K (20 mg/mL; Qiagen) was added to each reaction, followed by incubation for 7 min at 55°C. The samples were then analyzed by agarose gel electrophoresis. Typically all of the HMW DNA was converted to fragments of average size 400–500 bp. An image of a typical activity assay is shown in Figure 1C.

### Tagmentation reaction and final PCR amplification using Nextera and Nextera XT DNA sample preparation kits

In order to be able to compare the performance of the commercial kits with in-house transposase, we carried out the tagmentation with both Nextera and Nextera XT DNA sample preparation kits. When using the Nextera kit, we started from 5 ng cDNA after the preamplification reaction and added 25 μL of 2× tagment DNA buffer (TD), 5 μL of amplicon tagment mix (ATM), in a final volume of 50 μL. The solution was incubated for 5 min at 55°C, followed by purification with DNA Clean & Concentrator-5 (Zymo Research) according to the Nextera user manual. Elution was performed with 25 μL of resuspension buffer (RSB) from the Nextera kit, and 20 μL was used for the final enrichment PCR along with 15 μL of Nextera PCR master mix (NPM), 5 μL of Index 1 primers (N7xx), 5 μL of Index 2 primers (N5xx), and 5 μL of PCR primer cocktail (PPC; composed of FC-121-1012 and FC-121-1011 primers; see Illumina website). The PCR program was as follows: 3 min at 72°C, 30 sec at 98°C, and then five cycles of 10 sec at 98°C, 30 sec at 63°C, and 3 min at 72°C. When using the Nextera XT DNA sample preparation kit, samples were diluted in order to take 1 μL of DNA corresponding to different amounts (500 pg, 100 pg, 50 pg, 10 pg, 1 pg, 0.1 pg). We then added 10 μL of 2× tagment DNA buffer (TD), 5 μL of amplicon tagment mix (ATM) and 4 μL nuclease-free water (Gibco) and incubated for 5 min at 55°C. We added 5 μL of neutralize tagment buffer (NT), vortexed the solution, spun it down, and incubated it at room temperature for 5 min. The whole volume (25 μL) was then used for limited-cycle enrichment PCR using the following protocol: 3 min at 72°C, 30 sec at 95°C, and then n cycles of 10 sec at 95°C, 30 sec at 55°C, 30 sec at 72°C, where “n” depends on the amount of DNA used in the tagmentation (500 pg, *n* = 10; 100 pg, *n* = 12; 50 pg, *n* = 13; 10 pg, *n* = 15; 1 pg, *n* = 18; 0.1 pg, *n* = 20).

### Tagmentation reaction and final PCR amplification using in-house Tn5 transposase

Variable amounts of preamplified cDNA (5 ng, 500 pg, 100 pg, 10 pg, 1 pg, and 0.1 pg) were used for the tagmentation reaction carried out with in-house Tn5. The protocol is slightly different according to the input DNA used in the tagmentation reaction, as detailed below:

- Input DNA > 1 ng (preferably 2–5 ng). One microliter of Tn5 and 4 μL of 5× TAPS-DMF buffer (defined above) were used for each 50 μL reaction.
- Input DNA < 1 ng. Four microliters of 40% w/v PEG MW > 4000, 4 μL of 5× TAPS buffer, and a variable amount of Tn5 (0.01–1 μL, recommended 0.1 μL) were used for each 20 μL reaction. We recommend equilibrating the PEG at room temperature or at 37°C before assembling the reaction in order to reduce its viscosity and pipetting errors. We also suggest preparing a master mix of all reagents (including Tn5) but the sample DNA, quickly vortex it, spin it down, and keep it on ice until needed.

In both cases the tagmentation reaction was incubated for 7 min at 55°C. When starting from 5 ng DNA or more, column purification with DNA Clean & Concentrator-5 (Zymo Research) can be performed as described for the Nextera kit. The DNA was eluted with 25 μL water or EB solution (10 mM Tris-HCl at pH 7.5), and the whole volume was used for enrichment PCR as described below. However, when the input used for tagmentation is limited (< 1 ng DNA), the Tn5 was stripped off from the tagmented DNA, adding 5 μL of 0.2% SDS. After 5 min incubation at room temperature, samples were put on ice, and the whole volume (25 μL) was then used for limited-cycle enrichment PCR. The PCR master mix included 10 μL of Fidelity Buffer (5×), 1.5 μL dNTPs (10 mM), 1 μL KAPA HiFi DNA polymerase (1 unit/μL, all three reagents from KAPA Biosystems), 9.5 μL water (Gibco), and 1 μL each of Index 1 (0.5 μM, N7xx), Index 2 (0.5 μM, N5xx), and FC-121-1012 + FC-121-1011 primers (10 μM, all primers from Biomers.net). The use of FC-121-1012 and FC-121-1011 primers was not necessary, and we observed that the final libraries had fewer primer-dimers when these primers were omitted. Sequences of all indices and primers, as well as the amplification protocol, were the same as with the Nextera XT DNA sample preparation kit (see above). When the tagmentation is performed on < 1 ng DNA, the Illumina indices are diluted 1:5 prior to use, with no decrease in PCR yield. Although all libraries used KAPA HiFi in the enrichment PCR, we also successfully obtained libraries with KAPA HiFi HotStart ReadyMix, indicating that a HotStart DNA polymerase can, to some extent, be activated by the gap filling reaction at 72°C. With every PCR cycle, the number of active molecules increases, but still the final yield was approximately half of what was obtained with a non-HotStart enzyme.

When using the Nextera and Nextera XT protocol or our in-house Tn5 with KAPA HiFi polymerase, the purification of the final library was performed with 50 μL Agencourt AMPure XP beads (1:1 ratio with DNA), and the final elution was done with 15 μL EB solution. Samples were loaded on a high-sensitivity DNA chip for the final quality check, while quantification was performed with Qubit high-sensitivity DNA kit (Invitrogen). Libraries were pooled for sequencing on the Illumina HiSeq 2000 instrument at a final concentration of 2 nM each.

### Alternative buffers for tagmentation

The buffers listed below were all found to be compatible with tagmentation (when starting with ≥1 ng DNA). In this study we performed all experiments using the TAPS-based buffer, mainly due to its buffering capacity upon heat (as described in the manuscript).

5× TAPS buffer (used in the manuscript): 50 mM TAPS-NaOH, 25 mM MgCl_2_ (pH 8.5) at 25°C.
5× TAPS-DMF buffer (used in the manuscript): 50 mM TAPS-NaOH, 25 mM MgCl_2_, 50% v/v DMF (pH 8.5) at 25°C.
5× Tris-DMF buffer: 50 mM Tris-HCl, 25 mM MgCl_2_, 50% v/v DMF (pH 8.0) at 25°C. This buffer is identical to the one used by Wang et al. (2013).
5× HEPES-DMF buffer: 50 mM HEPES-KOH, 25 mM MgCl_2_, 50% v/v DMF, pH 7.2 at 25°C.
5× TAPS-KOAc-DMF buffer: 50 mM TAPS-KOAc, 25 mM MgCl_2_, 50 mM KOAc, 50% v/v DMF (pH 8.3) at 25°C.

### Single-cell isolation and RNA-sequencing

Single cells were manually picked under the microscope after resuspension in a solution composed of a 1:1 ratio of PBS and TrypLE Express (Gibco). To make sure only single cells were collected, the solution was visually inspected under the microscope, and discarded if multiple cells were observed. The liquid volume was kept as low as possible, usually < 0.3 μL. Cells were then transferred to a 0.2-mL thin-wall PCR tube containing 2 μL of a mild hypotonic lysis buffer composed of 0.2% Triton X-100 (Sigma) and 2 units/μL of RNase inhibitor (Clontech). Cells already picked were kept on ice throughout the process or stored at −80°C if not used immediately. Alternatively, samples can be picked in a 4 μL solution, composed of 2 μL of 0.4% Triton X-100 (already containing RNase inhibitor), 1 μL dNTP mix (10 mM; Fermentas), and 1 μL SMARTer-dT_30_VN (Biomers.net). Single-cell RNA-seq libraries were generated according to a previously defined protocol (Picelli et al. 2014).

### Sequencing and sequence data analysis

All libraries were sequenced on an Illumina HiSeq 2000 according to standard procedures. Computational analyses were carried out as previously described (Picelli et al. 2013).

#### Tagmentation of DNA ladder

We used the GeneRuler 100-bp DNA ladder (Thermo Scientific) for the evaluation of tagmentation of short DNA fragments (Fig. 2C).

## Data access

Raw sequence reads and processed gene expression values from this study have been submitted to the NCBI Sequence Read Archive (SRA; http://www.ncbi.nlm.nih.gov/sra) under accession number SRP043417 and to the NCBI Gene Expression Omnibus (GEO; http://www.ncbi.nlm.nih.gov/geo) under accession number GSE58652, respectively.

## Supplementary Material

Supplemental Material
