# Supplementary material for: Tn5 transposase and tagmentation procedures for massively scaled sequencing projects
Source: Genome Res. 2014 Dec;24(12):2033–40. doi: 10.1101/gr.177881.114 (PMC4248319; doi:10.1101/gr.177881.114)
Supplement: Supplemental Material [file supp_24_12_2033__index.html]

Tn5 transposase and tagmentation procedures for massively scaled sequencing projects — Tn5 transposase and tagmentation procedures for massively scaled sequencing projects — Supplemental Material 

# Tn5 transposase and tagmentation procedures for massively scaled sequencing projects

## Supplemental Material

**Files in this Data Supplement:**

- Supplemental Information.pdf
