# Supplementary material for: Tn5 transposase and tagmentation procedures for massively scaled sequencing projects
Source: Genome Res. 2014 Dec;24(12):2033–40. doi: 10.1101/gr.177881.114 (PMC4248319; doi:10.1101/gr.177881.114)
Supplement: Supplemental Material [file supp_gr.177881.114_Supplemental_Information.pdf]

Supplemental information for

Tn5 transposase and tagmentation procedures for massively scaled sequencing projects

Simone Picelli[1], Åsa K. Björklund[1,2], Björn Reinius[1,2], Sven Sagasser[1,2],

Gösta Winberg[1,2] and Rickard Sandberg[1,2].

Supplemental Items:

Supplemental Methods: Complete sequence of Tn5 expression vector

Supplemental Fig 1. Plasmid map of pTXB1-Tn5

Supplemental Fig 2. Sequencing library characteristics

Supplemental Fig 3. Tagmentation sequence bias

Supplemental Fig 4. Library characteristics from ultra low DNA input amounts

Supplemental Fig 5. Library complexity at variable input amounts

Supplemental Table 1: Detailed list of reaction conditions in Figure 3

Supplemental Methods

Complete sequence of vector used for Tn5 production (see Supplemental Figure 1 for a Plasmid map).

```
>pTXB1-Tn5 [length=8079] [version=17-JUN-2014] [topology=circular]
Expression vector pTXB1-Tn5, complete sequence.
AACTACGTCAGGTGGCACTTTTCGGGGAAATGTGCGCGGAACCCCTATTTGTTTATTTTTCTAAATACA
TTCAAATATGTATCCGCTCATGAGACAATAACCCTGATAAATGCTTCAATAATATTGAAAAAGGAAGAG
TATGAGTATTCAACATTTCCGTGTCGCCCTTATTCCCTTTTTTGCGGCATTTTGCCTTCCTGTTTTTGC
TCACCCAGAAACGCTGGTGAAAGTAAAAGATGCTGAAGATCAGTTGGGTGCACGAGTGGGTTACATCGA
ACTGGATCTCAACAGCGGTAAGATCCTTGAGAGTTTTCGCCCCGAAGAACGTTCTCCAATGATGAGCAC
TTTTAAAGTTCTGCTATGTGGCGCGGTATTATCCCGTGTTGACGCCGGGCAAGAGCAACTCGGTCGCCG
CATACACTATTCTCAGAATGACTTGGTTGAGTACTCACCAGTCACAGAAAAGCATCTTACGGATGGCAT
GACAGTAAGAGAATTATGCAGTGCTGCCATAACCATGAGTGATAACACTGCGGCCAACTTACTTCTGAC
AACGATCGGAGGACCGAAGGAGCTAACCGCTTTTTTGCACAACATGGGGGATCATGTAACTCGCCTTGA
TCGTTGGGAACCGGAGCTGAATGAAGCCATACCAAACGACGAGCGTGACACCACGATGCCTGTAGCAAT
GGCAACAACGTTGCGCAAACTATTAACTGGCGAACTACTTACTCTAGCTTCCCGGCAACAATTAATAGA
CTGGATGGAGGCGGATAAAGTTGCAGGACCACTTCTGCGCTCGGCCCTTCCGGCTGGCTGGTTTATTGC
TGATAAATCTGGAGCCGGTGAGCGTGGGTCTCGCGGTATCATTGCAGCACTGGGGCCAGATGGTAAGCC
CTCCCGTATCGTAGTTATCTACACGACGGGGAGTCAGGCAACTATGGATGAACGAAATAGACAGATCGC
TGAGATAGGTGCCTCACTGATTAAGCATTGGTAACTGTCAGACCAAGTTTACTCATATATACTTTAGAT
TGATTTACCCCGGTTGATAATCAGAAAAGCCCCAAAAACAGGAAGATTGTATAAGCAAATATTTAAATT
GTAAACGTTAATATTTTGTTAAAATTCGCGTTAAATTTTTGTTAAATCAGCTCATTTTTTAACCAATAG
GCCGAAATCGGCAAAATCCCTTATAAATCAAAAGAATAGCCCGAGATAGGGTTGAGTGTTGTTCCAGTT
TGGAACAAGAGTCCACTATTAAAGAACGTGGACTCCAACGTCAAAGGGCGAAAAACCGTCTATCAGGGC
GATGGCCCACTACGTGAACCATCACCCAAATCAAGTTTTTTGGGGTCGAGGTGCCGTAAAGCACTAAAT
CGGAACCCTAAAGGGAGCCCCCGATTTAGAGCTTGACGGGGAAAGCCGGCGAACGTGGCGAGAAAGGAA
GGGAAGAAAGCGAAAGGAGCGGGCGCTAGGGCGCTGGCAAGTGTAGCGGTCACGCTGCGCGTAACCACC
ACACCCGCCGCGCTTAATGCGCCGCTACAGGGCGCGTAAAAGGATCTAGGTGAAGATCCTTTTTGATAA
TCTCATGACCAAAATCCCTTAACGTGAGTTTTCGTTCCACTGAGCGTCAGACCCCGTAGAAAAGATCAA
AGGATCTTCTTGAGATCCTTTTTTTCTGCGCGTAATCTGCTGCTTGCAAACAAAAAAACCACCGCTACC
AGCGGTGGTTTGTTTGCCGGATCAAGAGCTACCAACTCTTTTTCCGAAGGTAACTGGCTTCAGCAGAGC
GCAGATACCAAATACTGTCCTTCTAGTGTAGCCGTAGTTAGGCCACCACTTCAAGAACTCTGTAGCACC
GCCTACATACCTCGCTCTGCTAATCCTGTTACCAGTGGCTGCTGCCAGTGGCGATAAGTCGTGTCTTAC
CGGGTTGGACTCAAGACGATAGTTACCGGATAAGGCGCAGCGGTCGGGCTGAACGGGGGGTTCGTGCAC
ACAGCCCAGCTTGGAGCGAACGACCTACACCGAACTGAGATACCTACAGCGTGAGCTATGAGAAAGCGC
CACGCTTCCCGAAGGGAGAAAGGCGGACAGGTATCCGGTAAGCGGCAGGGTCGGAACAGGAGAGCGCAC
GAGGGAGCTTCCAGGGGGAAACGCCTGGTATCTTTATAGTCCTGTCGGGTTTCGCCACCTCTGACTTGA
GCGTCGATTTTTGTGATGCTCGTCAGGGGGGCGGAGCCTATGGAAAAACGCCAGCAACGCGGCCTTTTT
ACGGTTCCTGGCCTTTTGCTGGCCTTTTGCTCACATGTTCTTTCCTGCGTTATCCCCTGATTCTGTGGA
TAACCGTATTACCGCCTTTGAGTGAGCTGATACCGCTCGCCGCAGCCGAACGACCGAGCGCAGCGAGTC
AGTGAGCGAGGAAGCTATGGTGCACTCTCAGTACAATCTGCTCTGATGCCGCATAGTTAAGCCAGTATA
CACTCCGCTATCGCTACGTGACTGGGTCATGGCTGCGCCCCGACACCCGCCAACACCCGCTGACGCGCC
CTGACGGGCTTGTCTGCTCCCGGCATCCGCTTACAGACAAGCTGTGACCGTCTCCGGGAGCTGCATGTG
TCAGAGGTTTTCACCGTCATCACCGAAACGCGCGAGGCAGCTGCGGTAAAGCTCATCAGCGTGGTCGTG
CAGCGATTCACAGATGTCTGCCTGTTCATCCGCGTCCAGCTCGTTGAGTTTCTCCAGAAGCGTTAATGT
CTGGCTTCTGATAAAGCGGGCCATGTTAAGGGCGGTTTTTTCCTGTTTGGTCACTGATGCCTCCGTGTA
AGGGGGATTTCTGTTCATGGGGGTAATGATACCGATGAAACGAGAGAGGATGCTCACGATACGGGTTAC
TGATGATGAACATGCCCGGTTACTGGAACGTTGTGAGGGTAAACAACTGGCGGTATGGATGCGGCGGGA
CCAGAGAAAAATCACTCAGGGTCAATGCCAGCCGAACGCCAGCAAGACGTAGCCCAGCGCGTCGGCCGC
CATGCCGGCGATAATGGCCTGCTTCTCGCCGAAACGTTTGGTGGCGGGACCAGTGACGAAGGCTTGAGC
GAGGGCGTGCAAGATTCCGAATACCGCAAGCGACAGGCCGATCATCGTCGCGCTCCAGCGAAAGCGGTC
CTCGCCGAAAATGACCCAGAGCGCTGCCGGCACCTGTCCTACGAGTTGCATGATAAAGAAGACAGTCAT
AAGTGCGGCGACGATAGTCATGCCCCGCGCCCACCGGAAGGAGCTGACTGGGTTGAAGGCTCTCAAGGG
CATCGGTCGAGATCCCGGTGCCTAATGAGTGAGCTAACTTACATTAATTGCGTTGCGCTCACTGCCCGC
TTTCCAGTCGGGAAACCTGTCGTGCCAGCTGCATTAATGAATCGGCCAACGCGCGGGGAGAGGCGGTTT
GCGTATTGGGCGCCAGGGTGGTTTTTCTTTTCACCAGTGAGACGGGCAACAGCTGATTGCCCTTCACCG
CCTGGCCCTGAGAGAGTTGCAGCAAGCGGTCCACGCTGGTTTGCCCCAGCAGGCGAAAATCCTGTTTGA
TGGTGGTTAACGGCGGGATATAACATGAGCTGTCTTCGGTATCGTCGTATCCCACTACCGAGATATCCG
```

CACCAACGCGCAGCCCGGACTCGGTAATGGCGCGCATTGCGCCCAGCGCCATCTGATCGTTGGCAACCA
GCATCGCAGTGGGAACGATGCCCTCATTCAGCATTTGCATGGTTTGTTGAAAACCGGACATGGCACTCC
AGTCGCCTTCCCGTTCCGCTATCGGCTGAATTTGATTGCGAGTGAGATATTTATGCCAGCCAGCCAGAC
GCAGACGCGCCGAGACAGAACTTAATGGGCCCGCTAACAGCGCGATTTGCTGGTGACCCAATGCGACCA
GATGCTCCACGCCCAGTCGCGTACCGTCTTCATGGGAGAAAATAATACTGTTGATGGGTGTCTGGTCAG
AGACATCAAGAAATAACGCCGGAACATTAGTGCAGGCAGCTTCCACAGCAATGGCATCCTGGTCATCCA
GCGGATAGTTAATGATCAGCCCACTGACGCGTTGCGCGAGAAGATTGTGCACCGCCGCTTTACAGGCTT
CGACGCCGCTTCGTTCTACCATCGACACCACCACGCTGGCACCCAGTTGATCGGCGCGAGATTTAATCG
CCGCGACAATTTGCGACGGCGCGTGCAGGGCCAGACTGGAGGTGGCAACGCCAATCAGCAACGACTGTT
TGCCCGCCAGTTGTTGTGCCACGCGGTTGGGAATGTAATTCAGCTCCGCCATCGCCGCTTCCACTTTTT
CCCGCGTTTTCGCAGAAACGTGGCTGGCCTGGTTCACCACGCGGGAAACGGTCTGATAAGAGACACCGG
CATACTCTGCGACATCGTATAACGTTACTGGTTTCACATTCACCACCCTGAATTGACTCTCTTCCGGGC
GCTATCATGCCATACCGCGAAAGGTTTTGCGCCATTCGATGGTGTCCGGGATCTCGACGCTCTCCCTTA
TGCGACTCCTGCATTAGGAAGCAGCCCAGTAGTAGGTTGAGGCCGTTGAGCACCGCCGCCGCAAGGAAT
GGTGCATGCCGGCATGCCGCCCTTTCGTCTTCAAGAATTAATTCCCAATTCCCCAGGCATCAAATAAAA
CGAAAGGCTCAGTCGAAAGACTGGGCCTTTCGTTTTATCTGTTGTTTGTCGGTGAACGCTCTCCTGAGT
AGGACAAATCCGCCGGGAGCGGATTTGAACGTTGCGAAGCAACGGCCCGGAGGGTGGCGGGCAGGACGC
CCGCCATAAACTGCCAGGAATTAATTCCCCAGGCATCAAATAAAACGAAAGGCTCAGTCGAAAGACTGG
GCCTTTCGTTTTATCTGTTGTTTGTCGGTGAACGCTCTCCTGAGTAGGACAAATCCGCCGGGAGCGGAT
TTGAACGTTGCGAAGCAACGGCCCGGAGGGTGGCGGGCAGGACGCCCGCCATAAACTGCCAGGAATTAA
TTCCCCAGGCATCAAATAAAACGAAAGGCTCAGTCGAAAGACTGGGCCTTTCGTTTTATCTGTTGTTTG
TCGGTGAACGCTCTCCTGAGTAGGACAAATCCGCCGGGAGCGGATTTGAACGTTGCGAAGCAACGGCCC
GGAGGGTGGCGGGCAGGACGCCCGCCATAAACTGCCAGGAATTAATTCCCCAGGCATCAAATAAAACGA
AAGGCTCAGTCGAAAGACTGGGCCTTTCGTTTTATCTGTTGTTTGTCGGTGAACGCTCTCCTGAGTAGG
ACAAATCCGCCGGGAGCGGATTTGAACGTTGCGAAGCAACGGCCCGGAGGGTGGCGGGCAGGACGCCCG
CCATAAACTGCCAGGAATTAATTCCCCAGGCATCAAATAAAACGAAAGGCTCAGTCGAAAGACTGGGCC
TTTCGTTTTATCTGTTGTTTGTCGGTGAACGCTCTCCTGAGTAGGACAAATCCGCCGGGAGCGGATTTG
AACGTTGCGAAGCAACGGCCCGGAGGGTGGCGGGCAGGACGCCCGCCATAAACTGCCAGGAATTGGGGA
TCGGAATTAATTCCCGGTTTAAACCGGGGATCTCGATCCCGCGAAATTAATACGACTCACTATAGGGGA
ATTGTGAGCGGATAACAATTCCCCTCTAGATATTTTGTTTACTTTAAGAAGGAGATATACATATGATTA
CCAGTGCACTGCATCGTGCGGCGGATTGGGCGAAAAGCGTGTTTTCTAGTGCTGCGCTGGGTGATCCGC
GTCGTACCGCGCGTCTGGTGAATGTTGCGGCGCAACTGGCCAAATATAGCGGCAAAAGCATTACCATTA
GCAGCGAAGGCAGCAAAGCCATGCAGGAAGGCGCGTATCGTTTTATTCGTAATCCGAACGTGAGCGCGG
AAGCGATTCGTAAAGCGGGTGCCATGCAGACCGTGAAACTGGCCCAGGAATTTCCGGAACTGCTGGCAA
TTGAAGATACCACCTCTCTGAGCTATCGTCATCAGGTGGCGGAAGAACTGGGCAAACTGGGTAGCATTC
AGGATAAAAGCCGTGGTTGGTGGGTGCATAGCGTGCTGCTGCTGGAAGCGACCACCTTTCGTACCGTGG
GCCTGCTGCATCAAGAATGGTGGATGCGTCCGGATGATCCGGCGGATGCGGATGAAAAAGAAAGCGGCA
AATGGCTGGCCGCTGCTGCAACTTCGCGTCTGAGAATGGGCAGCATGATGAGCAACGTGATTGCGGTGT
GCGATCGTGAAGCGGATATTCATGCGTATCTGCAAGATAAACTGGCCCATAACGAACGTTTTGTGGTGC
GTAGCAAACATCCGCGTAAAGATGTGGAAAGCGGCCTGTATCTGTATGATCACCTGAAAAACCAGCCGG
AACTGGGCGGCTATCAGATTAGCATTCCGCAGAAAGGCGTGGTGGATAAACGTGGCAAACGTAAAAACC
GTCCGGCGCGTAAAGCGAGCCTGAGCCTGCGTAGCGGCCGTATTACCCTGAAACAGGGCAACATTACCC
TGAACGCGGTGCTGGCCGAAGAAATTAATCCGCCGAAAGGCGAAACCCCGCTGAAATGGCTGCTGCTGA
CCAGCGAGCCGGTGGAAAGTCTGGCCCAAGCGCTGCGTGTGATTGATATTTATACCCATCGTTGGCGCA
TTGAAGAATTTCACAAAGCGTGGAAAACGGGTGCGGGTGCGGAACGTCAGCGTATGGAAGAACCGGATA
ACCTGGAACGTATGGTGAGCATTCTGAGCTTTGTGGCGGTGCGTCTGCTGCAACTGCGTGAATCTTTTA
CTCCGCCGCAAGCACTGCGTGCGCAGGGCCTGCTGAAAGAAGCGGAACACGTTGAAAGCCAGAGCGCGG
AAACCGTGCTGACCCCGGATGAATGCCAACTGCTGGGCTATCTGGATAAAGGCAAACGCAAACGCAAAG
AAAAAGCGGGCAGCCTGCAATGGGCGTATATGGCGATTGCGCGTCTGGGCGGCTTTATGGATAGCAAAC
GTACCGGCATTGCGAGCTGGGGTGCGCTGTGGGAAGGTTGGGAAGCGCTGCAAAGCAAACTGGATGGCT
TTCTGGCCGCGAAAGACCTGATGGCGCAGGGCATTAAAATCTGCATCACGGGAGATGCACTAGTTGCCC
TACCCGAGGGCGAGTCGGTACGCATCGCCGACATCGTGCCGGGTGCGCGGCCCAACAGTGACAACGCCA
TCGACCTGAAAGTCCTTGACCGGCATGGCAATCCCGTGCTCGCCGACCGGCTGTTCCACTCCGGCGAGC
ATCCGGTGTACACGGTGCGTACGGTCGAAGGTCTGCGTGTGACGGGCACCGCGAACCACCCGTTGTTGT
GTTTGGTCGACGTCGCCGGGGTGCCGACCCTGCTGTGGAAGCTGATCGACGAAATCAAGCCGGGCGATT
ACGCGGTGATTCAACGCAGCGCATTCAGCGTCGACTGTGCAGGTTTTGCCCGCGGGAAACCCGAATTTG
CGCCCACAACCTACACAGTCGGCGTCCCTGGACTGGTGCGTTTCTTGGAAGCACACCACCGAGACCCGG
ACGCCCAAGCTATCGCCGACGAGCTGACCGACGGGCGGTTCTACTACGCGAAAGTCGCCAGTGTCACCG
ACGCCGGCGTGCAGCCGGTGTATAGCCTTCGTGTCGACACGGCAGACCACGCGTTTATCACGAACGGGT
TCGTCAGCCACGCTACTGGCCTCACCGGTCTGAACTCAGGCCTCACGACAATCCTGGTGTATCCGCTT
GGCAGGTCAACACAGCTTATACTGCGGGACAATTGGTCACATATAACGGCAAGACGTATAAATGTTTGC

AGCCCCACACCTCCTTGGCAGGATGGGAACCATCCAACGTTCCTGCCTTGTGGCAGCTTCAATGACTGC
AGGAAGGGGATCCGGCTGCTAACAAAGCCCGAAAGGAAGCTGAGTTGGCTGCTGCCACCGCTGAGCAAT
AACTAGCATAACCCCTTGGGGCCTCTAAACGGGTCTTGAGGGGTTTTTTGCTGAAAGGAGGAACTATAT
CCGGAT

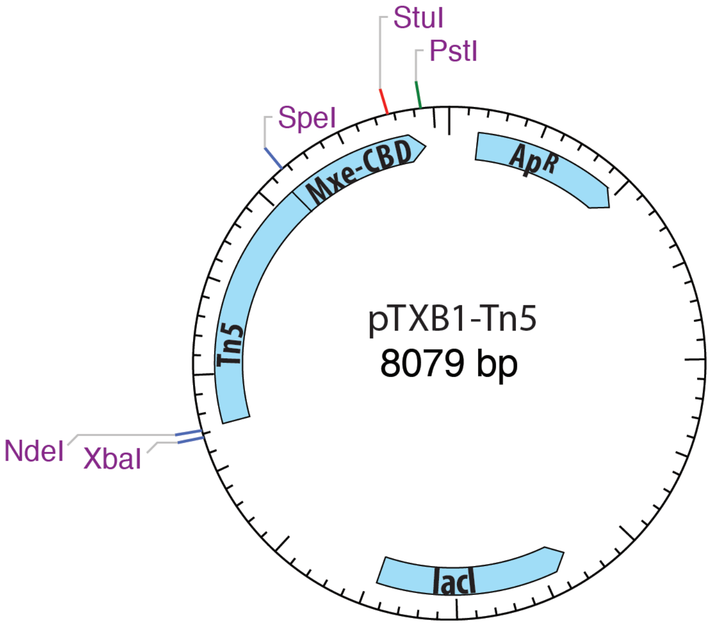

Supplemental Figure 1. Map of the pTXB1-Tn5 plasmid.

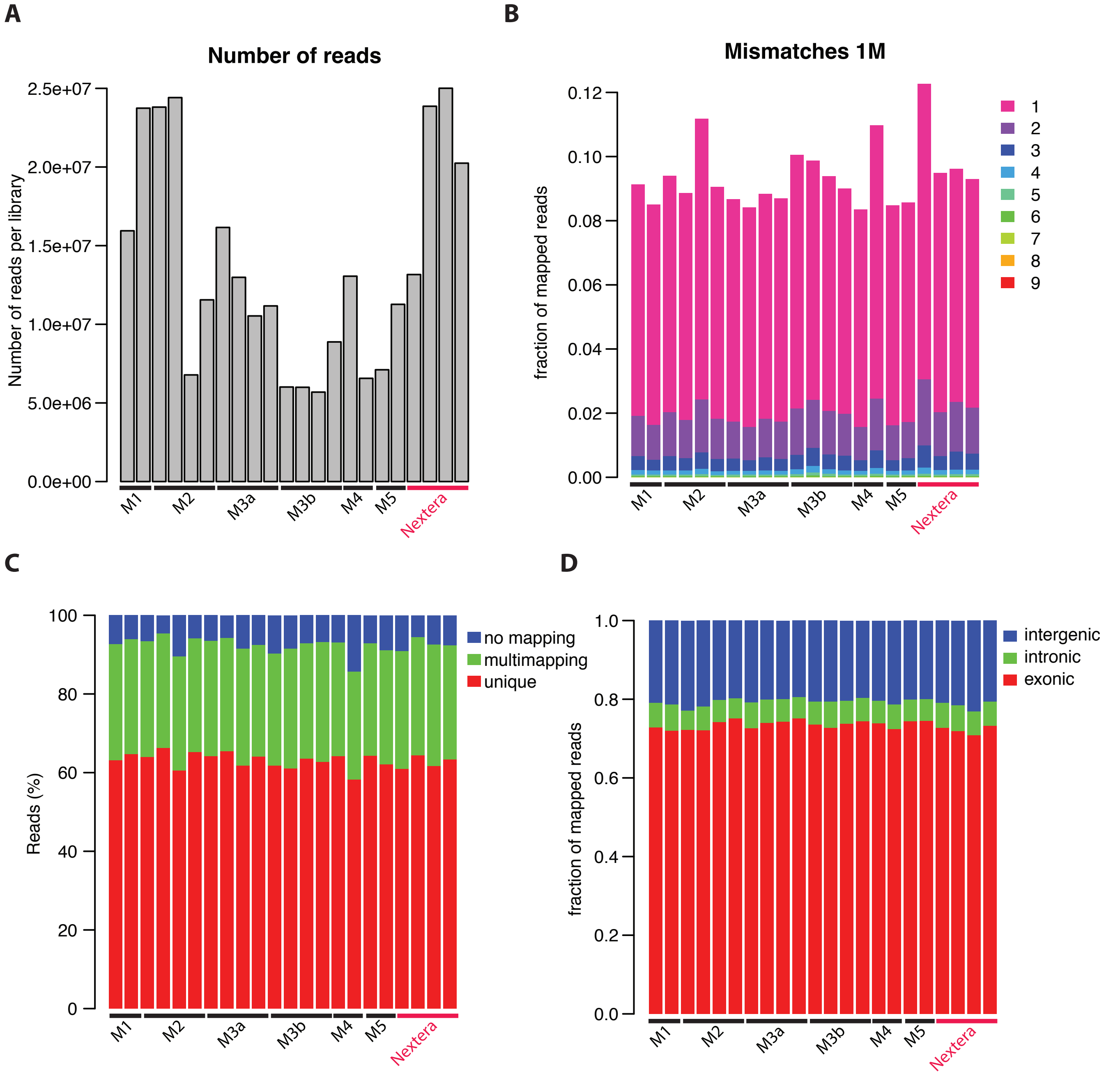

Supplemental Figure 2. Mapping statistics of RNA-seq libraries.
Libraries were generated with in-house or commercial Tn5 and reaction buffers as detailed in Supplemental Table 1. (**A**) Read depth per library. (**B**) Mismatch frequencies in libraries. (**C**) Percent reads mapping either uniquely to genome and transcriptome, to multiple locations (multimapping) or without any identifyable origin (no mapping). (**D**) Fraction of reads mapping to exonic, intronic or intergenic regions.

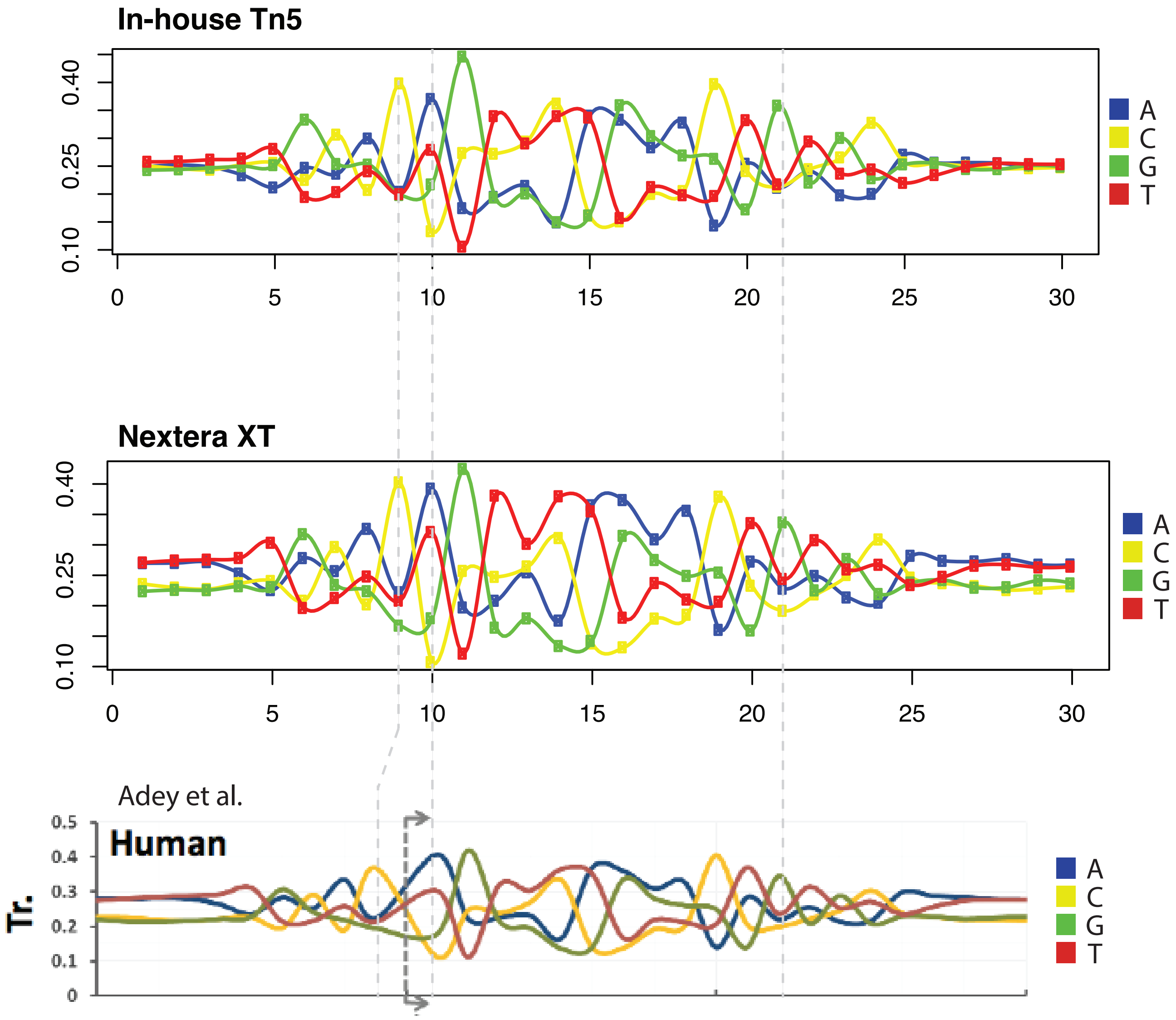

Supplemental Figure 3. Tagmentation sequence bias
Analyses of sequencing libraries generated with in-house Tn5 or Nextera XT demonstrate a bias in tagmentation sites very similar to that already described in Adey et al. 2010. The following two pages show bias estiamted from libraries generated from different starting amounts.

Supplemental Figure 3: continued

In-house Tn5 libraries

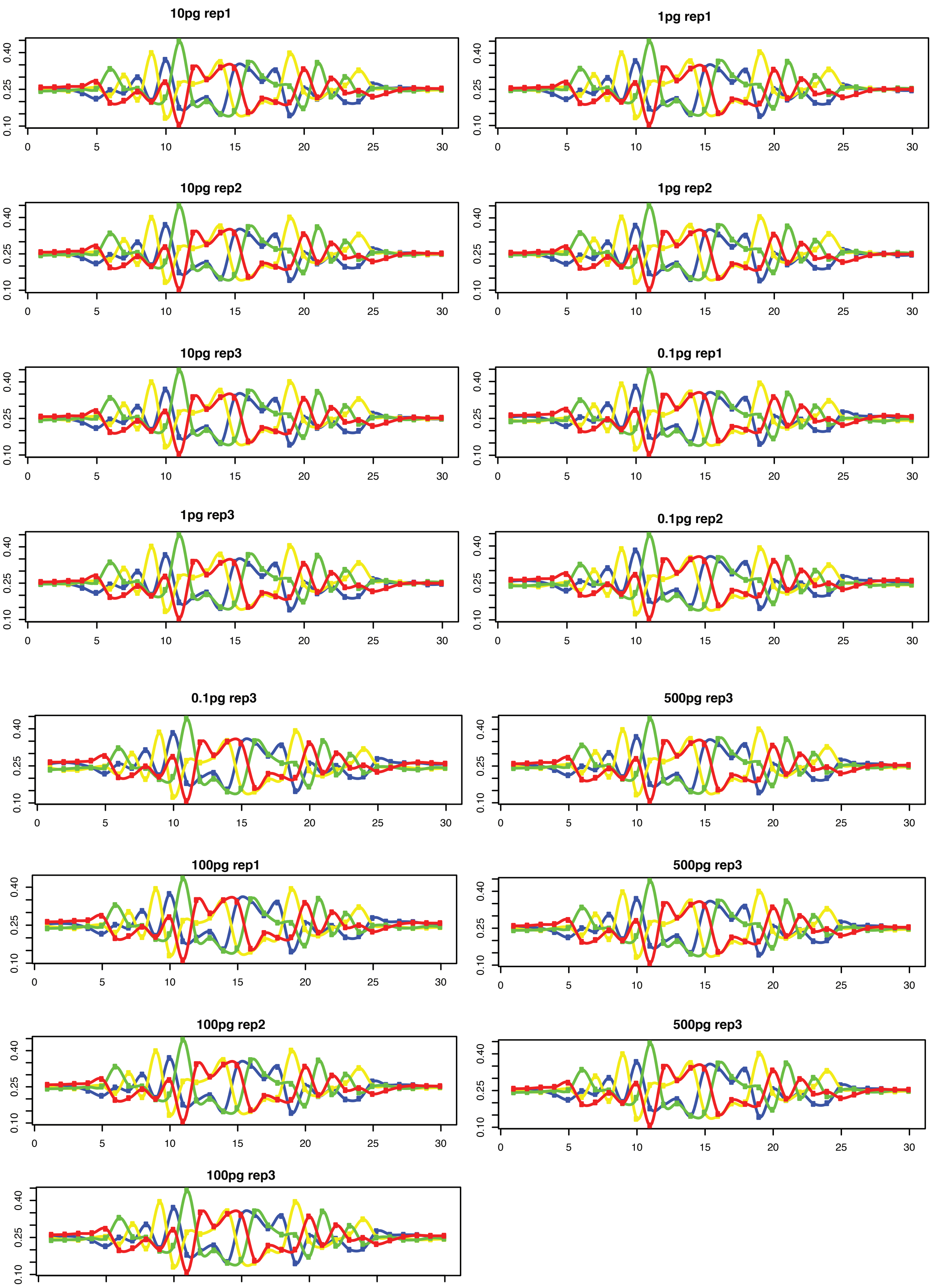

Supplemental Figure 3: continued

Nextera XT libraries

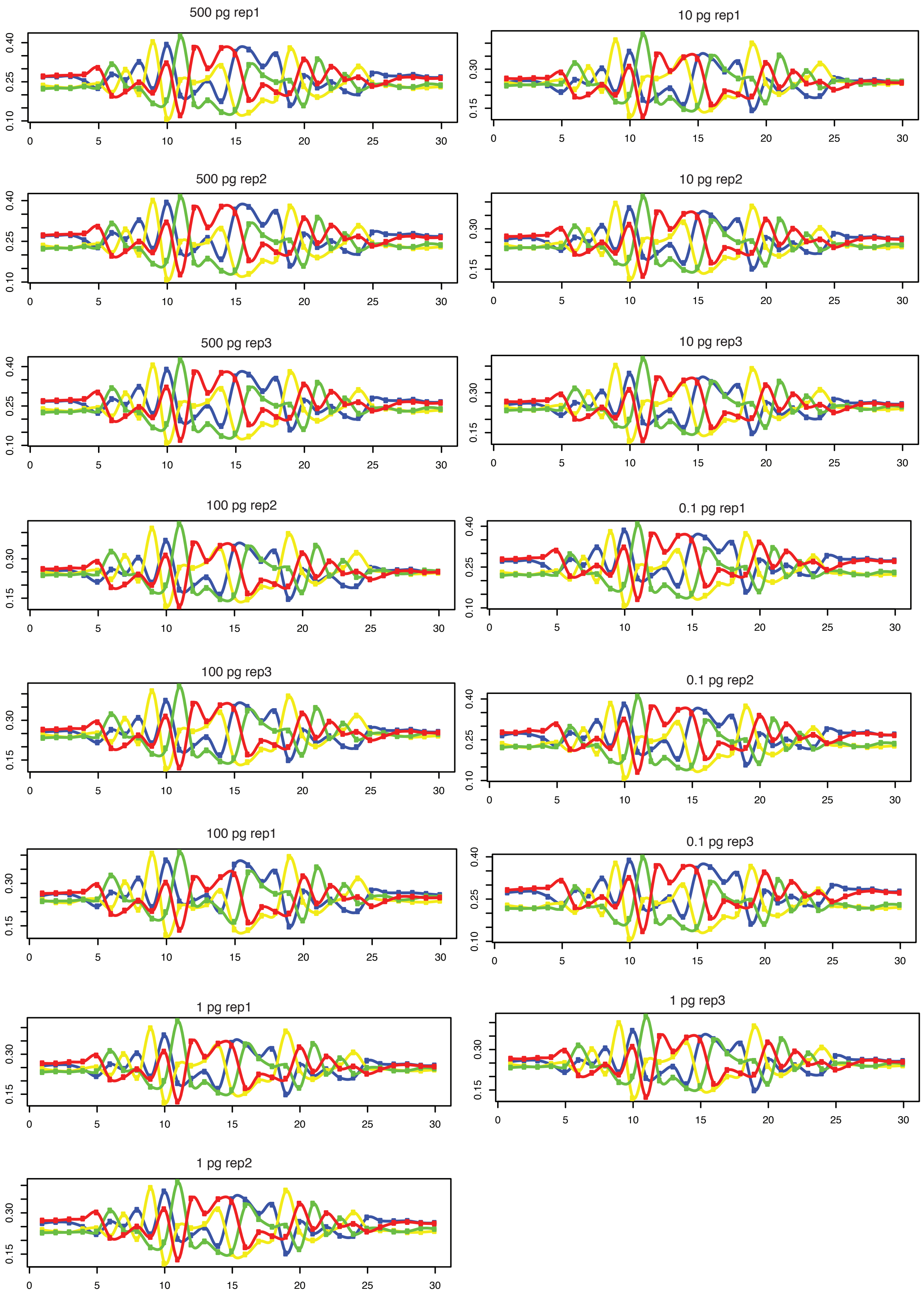

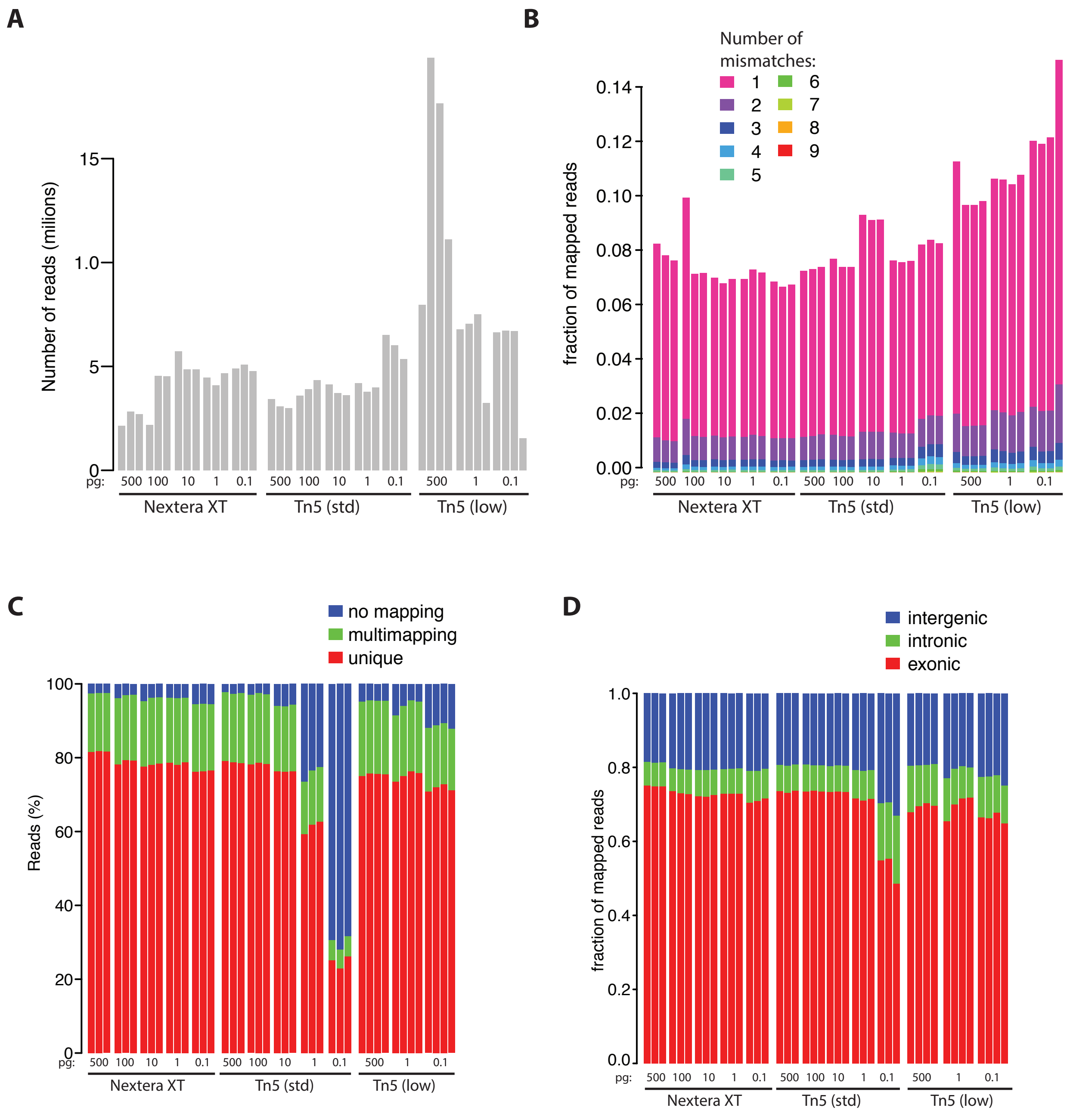

Supplemental Figure 4. Characteristics of libraries generated from ultra-low cDNA input. Libraries were generated with Nextera XT, in-house Tn5 and buffers using standard (std) or only a 1% of the amounts of Tn5 (low). Starting amounts in picogram cDNA is indicated below each graph. (**A**) Read depth per library. (**B**) Mismatch frequencies in libraries. (**C**) Percent reads mapping either uniquely to genome and transcriptome, to multiple locations (multimapping) or without any identifyable origin (no mapping). (**D**) Fraction of reads mapping to exonic, intronic or intergenic regions.

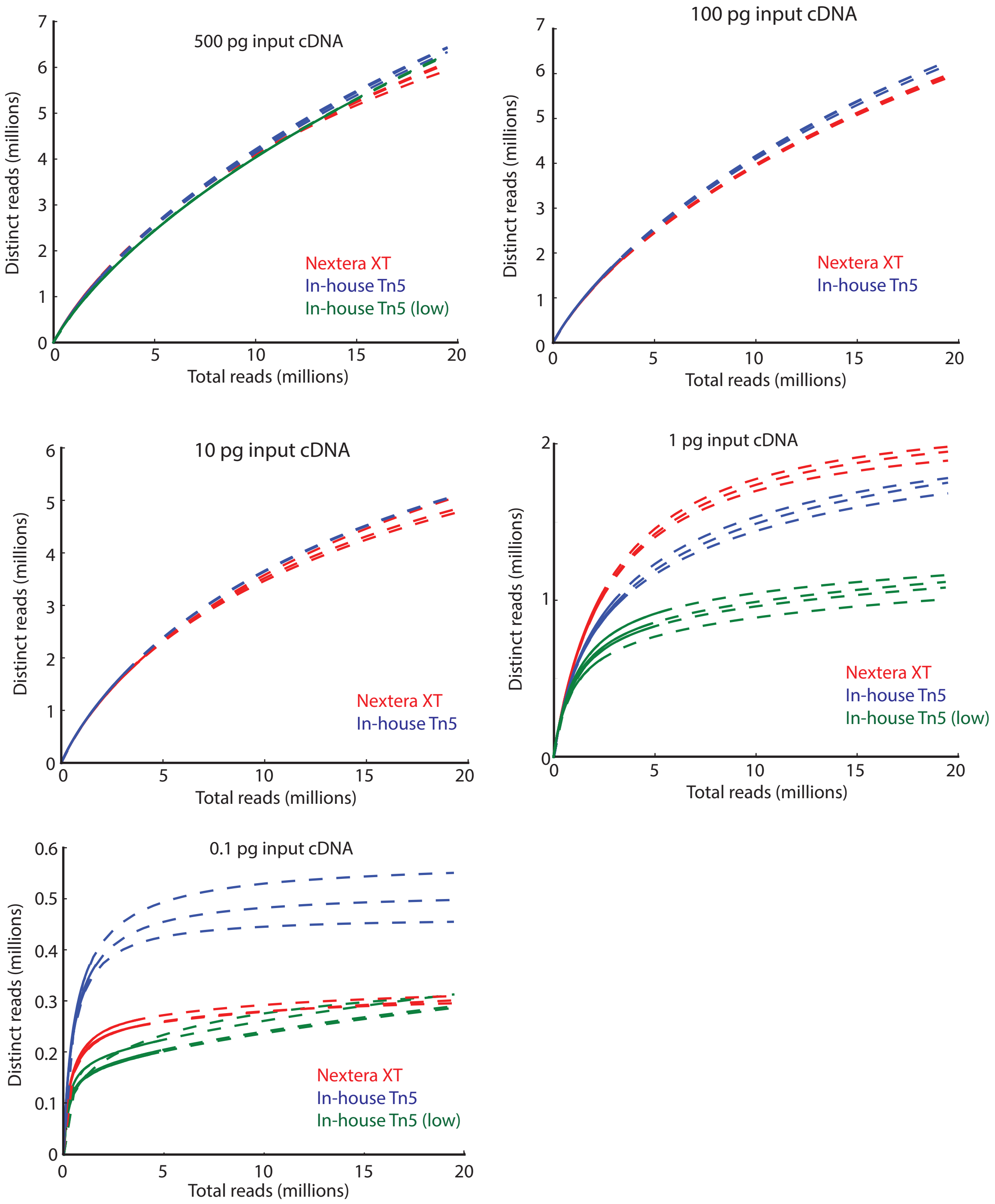

Supplemental Figure 5. Library complexity.
Library complexities were estimated using preseq (Daley et al. Nature Methods 2013). Solid lines represent subsampling of sequenced data and dashed lines extrapolations of the complexities in libraries if they were to be sequenced deeper.

Supplemental Table 1. Detailed list of reaction conditions compared in Fig 3.

Name	Tagmentation reaction volume (µl)	Tn5 transposase	Tagmentation buffer (5X)	Purification after tagmentation	Enrichment PCR	Figure
P1 (Standard Nextera®)	50	Nextera®-provided	Nextera® kit	Columns (DNA Clean & Concentrator)	Nextera® kit	Fig 3A,B
Modified 1	20	Nextera®-provided	Nextera® kit	5 µl NT buffer	Nextera® XT kit	Fig 3A,B
Modified 2	20	Nextera®-provided	Nextera® kit	5 µl NT buffer	KAPA HiFi DNA Polymerase	Fig 3A,B
Modified 3a	20	Nextera®-provided	Nextera® kit	0.01% SDS	KAPA HiFi DNA Polymerase	Fig 3A,B,C,D
Modified 4	20	Nextera®-provided	Nextera® kit	0.001% SDS	KAPA HiFi DNA Polymerase	Fig 3A,B
Modified 5	20	Nextera®-provided	Nextera® kit	ddH_2O	KAPA HiFi DNA Polymerase	Fig 3A,B
Modified 3b	20	In-house Tn5	50 mM TAPS-NaOH pH 8.5, 25 mM $MgCl_2$, 50% DMF	0.01% SDS	KAPA HiFi DNA Polymerase	Fig 3C,D
